# Sustainable HPLC method for simultaneous determination of Cefazolin, Sulfadimidine, and Marbofloxacin residues in milk

**DOI:** 10.1038/s41598-025-32613-7

**Published:** 2026-01-06

**Authors:** Sherin F. Hammad, Mahmoud Rabee, Rawda A. Abozeid, Samar H. Elagamy

**Affiliations:** 1 Faculty of Pharmacy, Department of Pharmaceutical Analytical Chemistry, Tanta University, Tanta, Egypt; 2 Research and Development department, Heliopolis University, Cairo, 11785 Egypt

**Keywords:** Milk, Cefazolin, Sulfadimidine, Marbofloxacin, Chromatography, Biochemistry, Biological techniques, Biotechnology, Chemistry, Environmental sciences

## Abstract

Antibiotic residues in milk pose a food safety risk by promoting antimicrobial resistance and causing toxic effects in consumers. This study developed and optimized a sensitive chromatographic method for the simultaneous quantification of Cefazolin (CFZ), Sulfadimidine (SDD), and Marbofloxacin (MFC) residues in milk. These antibiotics are commonly used as a combination therapy for managing cattle metritis. Separation was performed on a C18 column with UV detection at 270 nm. The eluent consisted of a 0.05 M phosphate buffer (pH 3), acetonitrile, and methanol in a 60:35:5 ratio, flowing at 1.0 mL/min. Sample preparation included protein precipitation with acetonitrile, followed by dispersive solid-phase extraction (dSPE) using Enhanced Matrix Removal-Lipid (EMR-L) tubes to remove interfering lipids. Validation per ICH-Q2R1 showed a linearity range of 0.0125–0.5 µg/mL for CFZ and MFC and 0.025–0.5 µg/mL for SDD, with LOD/LOQ values between 0.01 and 0.015 µg/mL and % recoveries between 97.67% and 98.89%. The method effectively quantified the studied drug residues in real cattle milk samples after their withdrawal period, with no significant differences in accuracy or precision versus established methods. Additionally, an assessment of the method’s sustainability (Eco-scale: 71, AGREE: 0.69, RGB12: 88.8) confirmed its compliance with green and white analytical chemistry principles.

**Supplementary Information:**

The online version contains supplementary material available at 10.1038/s41598-025-32613-7.

## Introduction

The presence of veterinary drug residues in animal-derived foods, such as meat, milk, eggs, and honey, can pose significant health risks to consumers. These risks include antimicrobial resistance, allergic reactions, potential carcinogenic or mutagenic effects, teratogenicity, and disturbances in gut microbiome^1^. The occurrence of these residues depends on various factors, such as the drug’s chemical properties, pharmacokinetic behavior, physiological characteristics of the animal, and biological processes involved in drug metabolism and elimination^2^. Failure to adhere to proper drug administration guidelines and disregarding withdrawal periods are also key factors contributing to veterinary drug residues in animal products^3^.

To mitigate these concerns, the World Health Organization (WHO) and global authorities have introduced strict laws to control the use and sale of veterinary medicines. These regulations set maximum residue limits (MRLs) for veterinary drugs in various food items. This enforcement strategy has shown partial success, cutting the legal distribution and sales of approved veterinary drugs for livestock by 43% between 2015 and 2016, followed by a further 33% drop from 2016 to 2017. Therefore, controlling MRLs is considered the first line of defense in reducing the presence and adverse health effects of these residues, which result from the high consumption of livestock products^4,5^.

Controlling veterinary drug residues in middle- and low-income countries presents significant challenges. Small farms often misuse and fail to comply with veterinary drug guidelines due to limited awareness among farmers. Regulatory efforts to monitor numerous small farms are hindered by resource constraints in countries with limited income^6^. The determination of multi-class, multi-residue veterinary drug residues primarily relies on liquid chromatography coupled with tandem mass spectrometry (LC-MS/MS)^7–10^. However, due to the high cost and limited availability of MS/MS instrumentation, many regulatory laboratories in low-income countries, such as Egypt, face challenges in implementing this analytical approach.

Antibiotics are extensively utilized in veterinary medicine, particularly in agriculture and aquaculture, accounting for a substantial share of the animal pharmaceutical market^11^. This research focuses on analyzing 3 commonly used antibiotics in milk samples: Cefazolin (CFZ), Sulfadimidine (SDD), and Marbofloxacin (MFC). These drugs are used together as a combination therapy for the management of metritis in cattle. Metritis is a uterine infection occurring within two weeks after calving. It is characterized by inflammation of the uterine wall and is often accompanied by foul-smelling, reddish-brown uterine discharge. This disease is typically caused by bacterial infection introduced during or shortly after parturition, and it can significantly affect reproductive performance and milk production in affected cattle. The selected antibiotics are commonly used together in this condition due to their proven efficacy and broad-spectrum activity. They have different mechanisms of action that may reduce resistance development, enhance bactericidal effects, and allow for dose optimization to minimize side effects^12–14^. These drugs are excreted unchanged in milk, and their residues can pose potential health risks to consumers^15–17^. The European Union (EU) has set maximum residue limits (MRL) for CFZ, SDD, and MFC in milk at 50, 100, and 150 µg/kg (equivalent to 0.05, 0.1, and 0.15 µg/mL), respectively^18,19^.

**Fig. 1:**
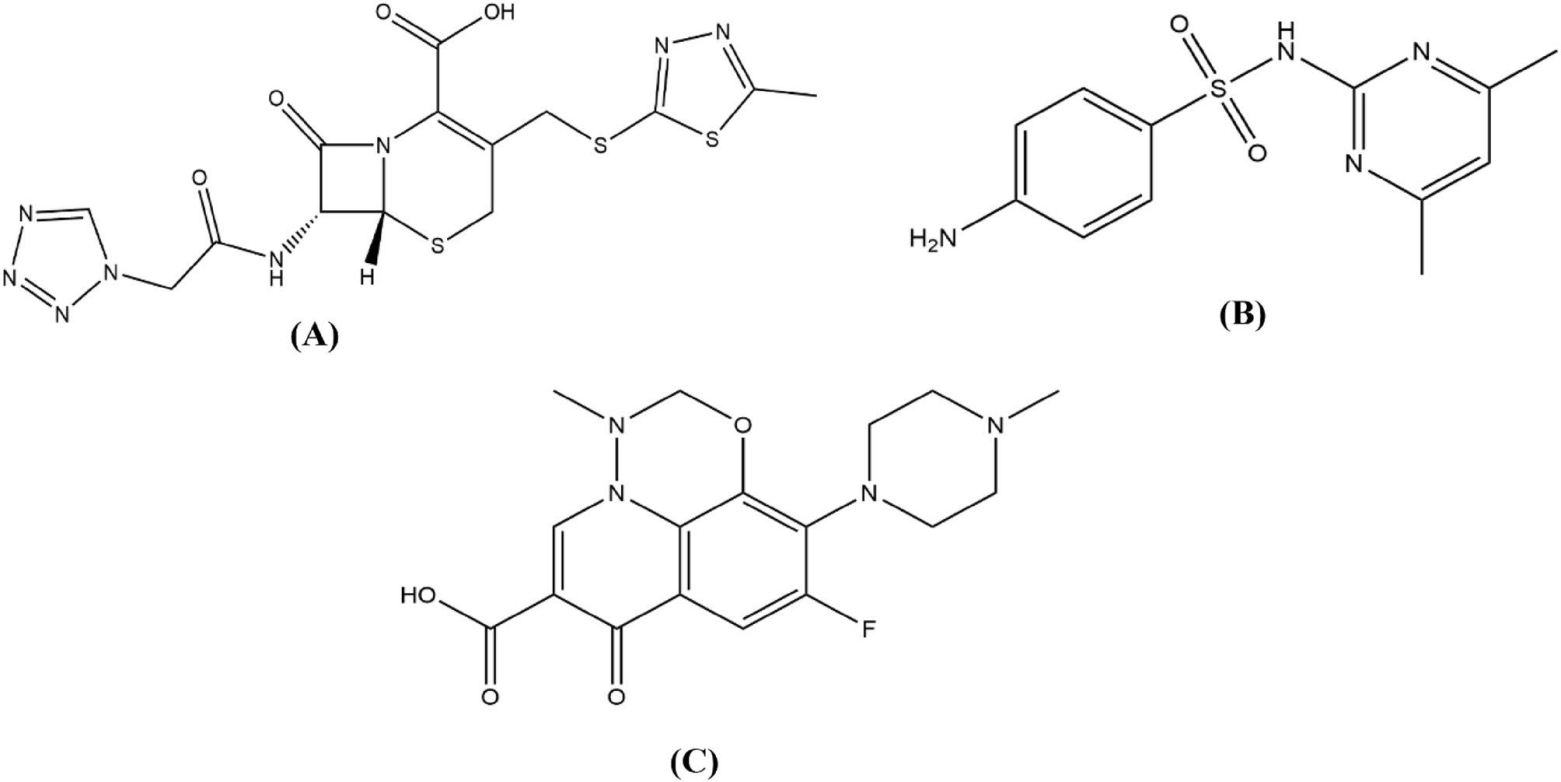
Chemical structures of (**A**) CFZ, (**B**) SDD, and (**C**) MFC.

**CFZ (**Fig. 1a**)** is a cephalosporin antibiotic mainly prescribed for bacterial skin infections and other serious systemic infections, affecting areas such as the lungs, bones, joints, stomach, bloodstream, heart valves, and urinary tract^20^. This bactericidal antibiotic works by blocking bacterial cell wall synthesis, mainly targeting Gram-positive aerobic bacteria. However, it has limited activity against Gram-negative bacteria and poor activity against anaerobes. CFZ is only available in injectable form for veterinary use and is typically administered at dosages ranging from 15 to 35 mg/kg. The dosage is given intravenously, intramuscularly, or subcutaneously every 6 to 8 h, depending on the type of infection and the response to treatment. CFZ is excreted unchanged in milk with a withdrawal period of 72 h^15^. Various methods have been reported for determining CFZ levels in milk, including TLC^21,22^, HPLC^15,23–27^, and LC-MS/MS^28–36^.

**SDD (**Fig. 1b**)**, chemically known as 4-amino-N-(4, 6-dimethyl-2-pyrimidinyl) benzene sulfonamide, is also referred to as sulfamethazine. This broad-spectrum sulfonamide antibiotic is widely used in veterinary medicine to treat bacterial and protozoal infections in livestock. It is administered via injection or orally in various formulations and requires careful dosing and treatment duration to ensure efficacy and reduce resistance development^37^. SDD is excreted unchanged in milk with a withdrawal period of 72 h^16^. Several analytical techniques have been reported for the determination of SDD in milk, including TLC^38,39^, LC-MS/MS^40–49^, and HPLC^41–49^.

**MFC (**Fig. 1c), a fluoroquinolone antibiotic, is widely employed in veterinary practice for treating diverse bacterial infections in animals. Its broad-spectrum efficacy makes it particularly useful against skin and soft tissue infections, UTIs, respiratory infections, and other bacterial diseases. MFC is available as injectable formulations and the typical dose is about 2 mg/kg once daily for 3–5 days^50^. It is excreted unchanged in milk with a withdrawal period of 72 h^17^. Several analytical methods have been reported for the determination of MFC in milk, such as HPLC^17,50–57^, LC-MS/MS^58–64^, and immunoassay determination^65–70^.

After reviewing the literature, it was found that LC-MS/MS methods have been developed for the determination of these antibiotics in combination with other antibiotic classes in feed samples^71^ and in bovine, swine, and fish meat^72^; however, no method has been reported for their combined determination in milk. Therefore, the aim of this study is to develop a validated and sustainable HPLC method for their combined determination in milk.

Prior to analysis, it is important to prepare the sample to ensure accurate measurement of the analyte. Raw samples may contain interfering substances or be incompatible with the analytical instrument^73^. Traditional extraction methods like liquid-liquid extraction have limitations such as low selectivity, high solvent usage, and labor-intensive procedures. Protein precipitation extraction is a simpler and more cost-effective alternative that can be effective for complex matrices like milk^74^. However, the high lipid content in milk often requires an additional cleanup step to remove fats from the extracts after protein precipitation. The dispersive solid phase extraction technique is now commonly used to minimize matrix effects from milk fats and achieve good recoveries without lengthy procedures^75^. Agilent’s Enhanced Matrix Removal-Lipid (EMR-Lipid) has been utilized for dispersive solid phase extraction since 2016. This technology efficiently removes lipids from complex samples without compromising analyte recovery. By using a unique sorbent material that combines size exclusion and hydrophobic interactions, EMR-Lipid selectively targets and eliminates lipids while preserving the analytes of interest. This advancement in sample cleanup for lipid-rich samples results in cleaner extracts, higher-quality data, and improved laboratory efficiency while maintaining the analyte integrity^76^.

This research introduces a green, high-sensitivity HPLC technique for detecting and quantifying the target drug residues in milk. The proposed method was applied to quantify the studied drug residues in cattle milk samples after their withdrawal period. The approach was validated following ICH Q2 (R1) standards, using protein precipitation and dispersive solid-phase extraction for sample cleanup. Its accuracy was confirmed by comparing results with standard reference methods. The method’s sustainability was also evaluated using Analytical Eco-Scale, AGREE, and RGB12 tools to ensure its optimal performance.

## Experimental

### Instruments

Chromatographic analysis was conducted with an Agilent 1100 HPLC system featuring a quaternary solvent delivery system (G1311A), automated sampler (G1329B), and programmable UV-Vis detector (G1314A). The stationary phase consisted of a C18 ODS Hypersil column (250 × 2.1 mm ID, 3 μm) operated under optimized parameters. Supporting instrumentation included a Bio-Base PHS-3BW precision pH meter and Bio-Base BTFC-12MRT3 high-speed centrifuge for sample preparation.

### Materials

#### Standards

The following pharmaceutical-grade reference standards were procured from Pharma-Swede Company (Egypt): CFZ (98.86% purity), SDD (99.37% purity), and MFC (99.99% purity). The certified purity levels were confirmed by the manufacturer’s certificates of analysis (CoA).

#### Milk samples

Five fresh raw milk samples were collected from five mature lactating cattle (3 buffalo and 2 cows). Their age and weight were approximately 2 years and 525 ± 28 kg, respectively. They were kept in a tie-stall housing system on a farm located in Sharqia Governorate, Egypt. The selected cattle were metritis-positive cases and were receiving the combination therapy of the studied drugs in their recommended doses by a veterinarian. The milk samples (25 mL) were collected by a veterinarian after the withdrawal period of the studied drugs (72 h) in sterile Falcon tubes (50 mL) and then stored at -20 °C until laboratory analysis. Prior permission was obtained from the farm owner. All procedures for sample collection were performed in compliance with institutional and national guidelines for the care and use of animals. Further details regarding ethical approval and informed consent are provided in the *Ethics approval and consent to participate* section. All animal experiments in this study were designed, conducted, and reported in accordance with the ARRIVE guidelines^77^. Before analysis, the samples were filtered through 0.45-µm nylon membrane filters to eliminate the suspended matter. During the method development and validation, drug-free milk (organic) was used to ensure the absence of any other drug residues that may cause potential matrix effects.

#### Chemicals & reagents

All chemicals used were HPLC-grade from reputable sources: acetonitrile and methanol (Honeywell), purified water (LiChrosolv), potassium dihydrogen phosphate (Sigma-Aldrich), plus glacial acetic acid and phosphoric acid (EL-NASR Chemicals). For the dispersive solid-phase extraction procedure, Bond Elut EMR-Lipid d-SPE tubes (1.0 g in 15 ml tubes, p/n: 5982 − 1010) were obtained from Agilent Technologies (Germany). All chemicals met the required purity standards for chromatographic analysis.

#### Standard solutions

The solubility of CFZ, SDD, and MFC in water was 100, 0.5, and 0.7 mg/mL, respectively^15–17^. Stock standards of CFZ, SDD, and MFC (25 mg each) were dissolved in 250 mL water to yield 100 µg/mL stock solutions. Daily working standards (10 µg/mL) were freshly prepared by diluting 5 mL stock solution to 50 mL with water.

### Procedures

#### Samples preparation

**Fig. 2:**
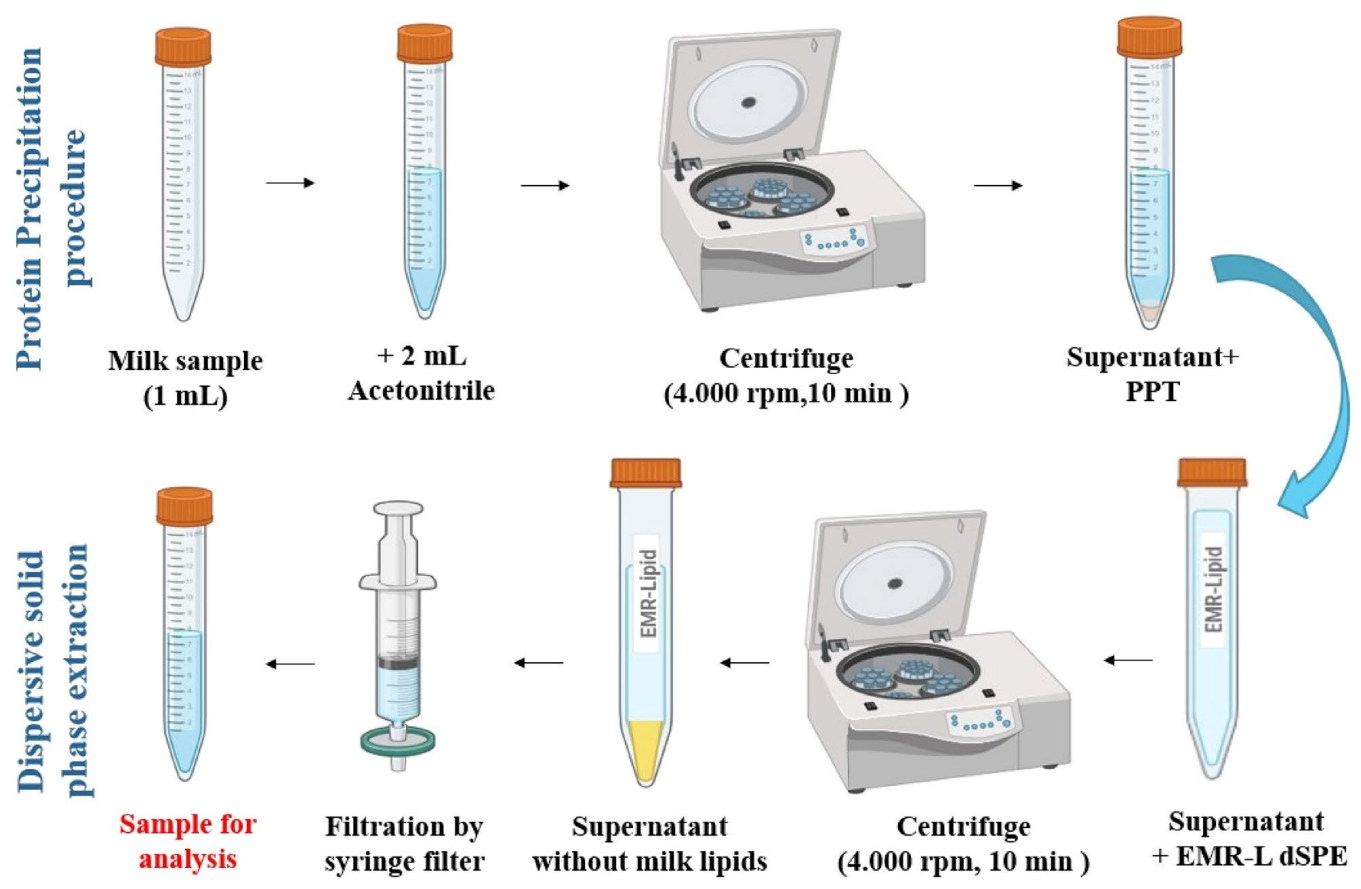
The flow chart of the sample preparation procedure.

In a 10 mL centrifugation tube, 1.0 mL (equivalent to 1 g) of milk sample was mixed with 2.0 mL of acetonitrile to induce precipitation of milk proteins. The mixture was then centrifuged at 4,000 rpm for 10 min. The resulting supernatant was transferred to a 15-mL EMR-Lipid dispersive solid-phase extraction tube to eliminate milk fats. The activated EMR-Lipid dSPE tube was centrifuged at 4,000 rpm for 10 min. The clear supernatant was then filtered and transferred into a clean tube. The prepared sample was subsequently subjected to chromatographic analysis under the optimized conditions. The sample preparation procedure is detailed in Fig. 2.

#### Chromatographic conditions

The chromatographic analysis was performed using a reversed-phase ODS Hypersil C18 column (250 × 2.1 mm, 3 μm). The analysis used an isocratic mobile phase of pH 3 phosphate buffer, acetonitrile and methanol (60:35:5, v/v/v) flowing at 1 mL/min. UV detection at 270 nm was performed with 50 µL injections at room temperature.

#### Validation of the procedures

##### Construction of calibration curves (Linearity)

Standard solutions were prepared by transferring measured volumes (0.1-5 mL) from the 10 µg/mL stock solutions of CFZ, SDD, and MFC into 100 mL volumetric flasks. After dilution to the mark with distilled water and complete mixing, the resulting solutions covered a concentration range of 0.01–0.5 µg/mL. This concentration range was across the specified MRL of each drug. The MRLs for CFZ, SDD, and MFC are 0.05, 0.1, and 0.15 µg/mL, respectively. These solutions were prepared three times and injected in triplicate (50 µL each) into the HPLC system. Drug concentrations were calculated using linear calibration plots of peak area (270 nm) versus concentration, with regression equations applied to all test samples.

##### System suitability parameters

The analytical performance of the chromatographic method was rigorously assessed through comprehensive evaluation of key parameters: retention time (tR), peak resolution (Rs), peak symmetry/tailing factor (T), retention capacity factor (k’), separation selectivity factor (α), column efficiency (N), and the height equivalent to a theoretical plate (HETP). All calculations were performed in accordance with ICH Q2 (R1) validation requirements to ensure method reliability prior to routine analytical application.

##### Accuracy and precision

The analytical procedure’s trueness (accuracy) and repeatability (precision) were assessed through triplicate measurements at three concentration levels (0.05, 0.15, and 0.25 µg/mL) for each target compound. Intra-day variability was determined by replicate analyses performed during a single analytical run, whereas inter-day reproducibility was evaluated over three separate analytical sessions conducted on consecutive days. Method accuracy was quantified as the percentage recovery with associated standard deviation, while measurement precision was characterized using the coefficient of variation (RSD %).

##### Evaluation of analyte extraction efficiency

The extraction efficiency/recovery for the target analytes was assessed by measuring the average chromatographic peak areas from milk samples prepared at three spiked levels (0.05, 0.15, and 0.25 µg/mL) and comparing these responses after extraction with those obtained from water standard solutions of equivalent concentrations. While complete (100%) recovery is not required, the extraction procedure must demonstrate consistent and reproducible analyte recuperation across all tested concentration ranges, with acceptable precision in the measured recoveries.

##### Limits of detection and quantitation

The limits of detection (LOD) and quantification (LOQ) were determined in accordance with ICH Q2(R1) recommendations through statistical treatment of calibration data, employing the mathematical expressions: LOD = 3.3σ/S and LOQ = 10σ/S, where σ corresponds to the standard deviation of the y-intercept values derived from linear regression analysis and S denotes the slope of the calibration curve.

##### Specificity and selectivity

Five randomly selected drug-free milk samples were processed using the same described sample preparation procedure and analyzed under the specified chromatographic conditions to determine the extent to which the endogenous milk components may interfere with the studied drugs. The selected drug-free milk samples were from different types of milk, including three raw buffalo milk samples, two raw cow milk samples, and one raw goat milk sample.

##### Application on real milk samples

Milk samples were collected individually from five lactating, metritis-positive cattle after the withdrawal period for each drug. The withdrawal period is the required time between the last administration of a veterinary medicine and the slaughter or food production from that animal^6^. Based on the literature, the withdrawal period for CFZ, SDD, and MFC in milk was approximately 72 h^15–17^. The last dose of the drugs was administered intramuscularly to the affected cattle simultaneously by a veterinarian. Subsequently, five milk samples (25 mL each) were collected after 72 h, processed, and analyzed using the specified chromatographic conditions. The concentrations in µg/mL were determined from the respective regression equations for each drug and compared to the Maximum Residual Limit (MRL) for each drug.

## Result and discussion

This study aimed to develop a reliable and precise chromatographic method for the simultaneous quantification and routine quality control of CFZ, SDD, and MFC residues in milk samples. To ensure accurate determination, we optimized the extraction process and mobile phase composition, minimizing potential interference from impurities.

### Optimization of experimental conditions

**Fig. 3:**
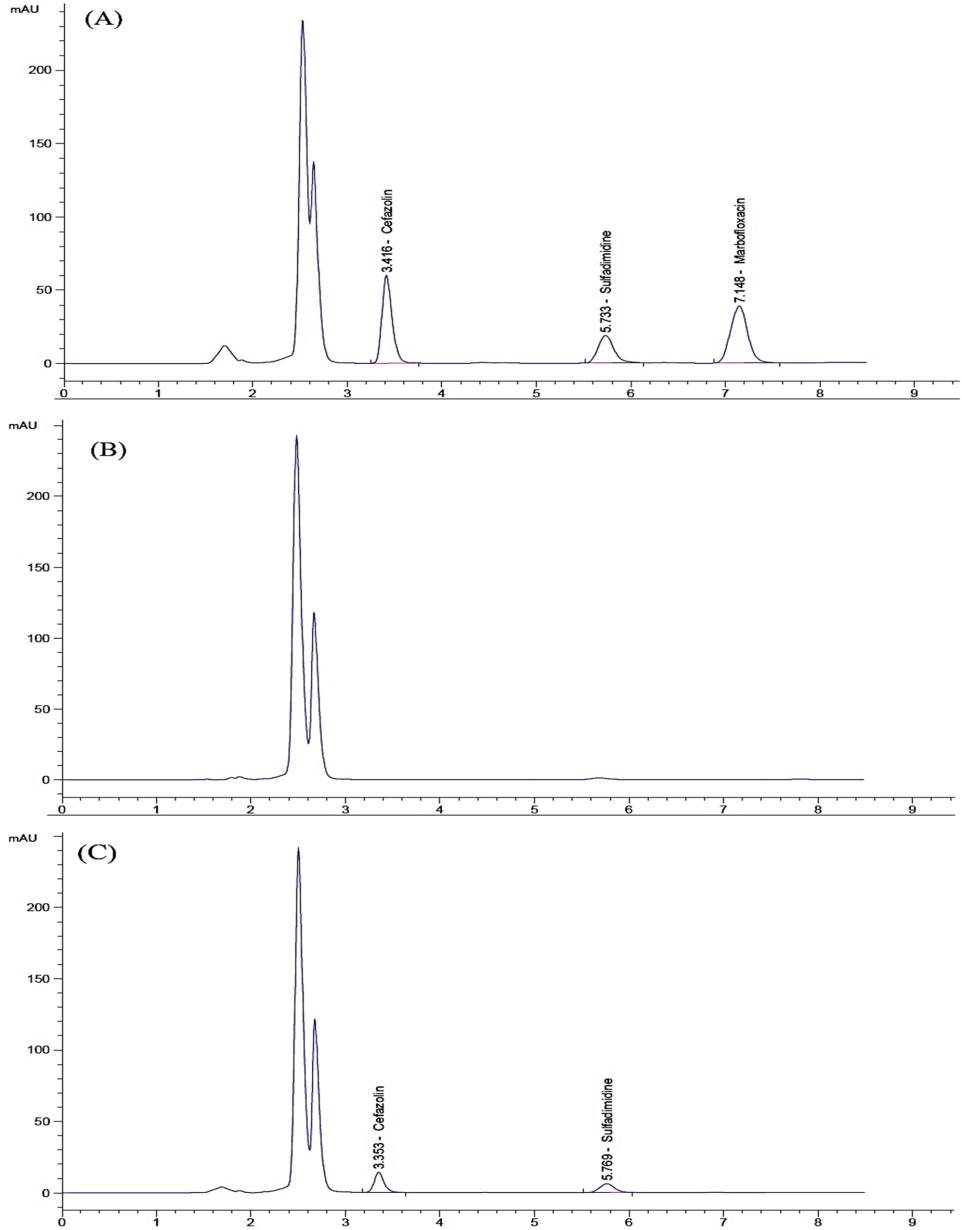
HPLC chromatograms of (**A**) milk sample spiked with the studied drugs (0.05 µg/mL each), (**B**) drug-free milk sample, and (**C**) real milk sample (no.1) showing CFZ and SDD residues.

The pKa values for CFZ, SDD, and MFC are 2.84, 7.5, and 5.69, respectively, while log P values are − 0.58, 0.19, and − 0.835, respectively^78–80^. The chromatographic separation was systematically optimized to achieve maximum resolution of the target analytes. Two stationary phase (C8 ODS Inertsil and C18 ODS Hypersil) were evaluated, with the C18 column demonstrating superior selectivity and peak resolution. To increase the signal intensity and method sensitivity to reach the MRL level for each drug, a C18 ODS Hypersil column (250 × 2.1 mm, 3 μm) was used instead of the classic C18 ODS Hypersil column (250 × 4.6 mm, 5 μm). Reducing the column’s internal diameter enhances sensitivity by concentrating the analyte into a smaller volume, yielding taller peaks and a better signal-to-noise ratio. Using a smaller particle size also increases sensitivity by improving the column efficiency, resulting in sharper, taller peaks and enhanced resolution. Increasing the injection volume also improves the method sensitivity by enhancing the signal intensity, but only up to a certain point where column capacity and resolution are not compromised. An injection volume of 50 µL was found to be suitable without any peak broadening or tailing for the studied drugs. Initial isocratic elution using a binary mobile phase (acetonitrile: water, 50:50 v/v) resulted in insufficient separation between analytes and matrix interferences. The introduction of methanol as a modifier significantly enhanced chromatographic efficiency, reducing the total run time to 10 min. Further optimization revealed that replacing water with 0.05 M phosphate buffer (pH 3.0) improved peak symmetry, resolution, and overall method robustness. The method optimization culminated in the implementation of a ternary solvent system comprising 0.05 M phosphate buffer (pH-adjusted to 3.0), acetonitrile, and methanol in a precisely controlled volumetric ratio of 60:35:5 (v/v/v). This chromatographic condition facilitated complete baseline resolution with optimal peak symmetry and sharpness within an 8-minute analytical run at the standardized flow rate of 1.0 mL/min. Experimental observations confirmed that any variation from this specified flow rate resulted in compromised separation efficiency, manifested through peak distortion, tailing phenomena, and elevated system backpressure. UV detection was performed at 270 nm, corresponding to the λmax of SDD while ensuring optimal sensitivity for CFZ and MFC (Figure S1). The method exhibited excellent reproducibility, with mean retention times (tR) of 3.36 ± 0.2 min (CFZ), 5.73 ± 0.2 min (SDD), and 6.93 ± 0.2 min (MFC), as illustrated in Fig. 3.

### System suitability parameters

A comprehensive system suitability evaluation was conducted to verify the performance characteristics of the developed chromatographic method according to International Council for Harmonization (ICH) standards. The assessment criteria encompassed critical chromatographic parameters including tR, Rs, T, K’, α, N, and HETP. The chromatographic peaks of CFZ, SDD, and MFC demonstrated excellent symmetry, with tailing factors (T) consistently near 1.0, indicating ideal peak shape. The selectivity factors (α) confirmed sufficient chromatographic separation, while resolution values (Rs) exceeded 1.5 for all analyte pairs, ensuring baseline separation. These results confirm that the chromatographic system meets the required performance criteria for the simultaneous analysis of these compounds. The complete system suitability data are presented in Table 1.

**Table 1:** Chromatographic performance characteristics established for the validation of the optimized HPLC method.

Parameters	CFZ	SDD	MFC	Reference value^84^
Retention time (t_R_) (min)	3.4 ± 0.2	5.7 ± 0.2	7.1 ± 0.2	–
Resolution (R_s_)^a^	–	9.59	4.24	> 1.5
Symmetry factor (T)	0.82	0.84	1.02	~ 1
Capacity factor (K)	1.94	3.25	4.63	1–10 acceptable
Selectivity factor (α)^a^	–	1.49	1.36	α > 1
Number of theoretical plates (N) = Column efficiency	11,254	10,264	12,551	≥ 2000, Increases with efficiency of separation
HETP = Height Equivalent Theoretical Plate (mm)	0.031	0.021	0.019	The smaller the value, The higher the column efficiency

### Method validation

The validation of the analytical method was performed following the International Council for Harmonization (ICH) Q2 (R1) recommendations^81^, with comprehensive assessment of key validation parameters such as linearity, specificity, accuracy, repeatability, and intermediate precision.

#### Linearity

The calibration curves for the target compounds were generated by establishing the relationship between the peak area (at 270 nm) and the corresponding drug concentrations (µg/mL) under the optimized chromatographic conditions. The regression plots were found to be linear over the range of 0.0125–0.5 µg/mL for both CFZ and MFC and 0.025–0.5 µg/mL for SDD. These linear concentration ranges were within the specified MRL of each drug. The MRLs for CFZ, SDD, and MFC are 0.05, 0.1, and 0.15 µg/mL, respectively. The complete analytical parameters, including the linear dynamic ranges, mathematical regression models, y-intercepts, calibration slopes, and correlation coefficients (r), are summarized in Table 2. The strong linear correlation is demonstrated by high correlation coefficient values across all calibration curves.

**Table 2:** Regression parameters for determination of Cefazolin (CFZ), sulfadimidine (SDD), and Marbofloxacin (MFC) by the proposed HPLC method.

Parameters	CFZ	SDD	MFC
Range (µg/mL)	0.0125–0.5	0.025–0.5	0.0125–0.5
MRL (µg/mL)	0.05	0.1	0.15
Wavelength (nm)	270	270	270
Regression Parameters Slope ± SD Intercept ± SD SD of residuals (Sy/x)	8220.061 ± 91.25	5363.671 ± 119.77	10511.042 ± 174.44
	-8.281 ± 5.03	-75.333 ± 18.69	-76.798 ± 27.72
	5729.779	6455.411	20937.019
Correlation coefficient (r)	0.9995	0.9992	0.9991
LOD (µg/mL)	0.01	0.015	0.01
LOQ (µg/mL)	0.012	0.02	0.012

#### Detection and quantitation limits

The analytical sensitivity of the developed method was assessed through the determination of the limit of detection (LOD) and the limit of quantitation (LOQ). These parameters were derived using the expressions **LOD = 3.3σ/S** and **LOQ = 10σ/S**, where **σ** corresponds to the standard deviation of the regression intercept, and **S** signifies the slope of the calibration curve. The calculated LOD and LOQ values, summarized in Table 2, underscore the high sensitivity achieved by the proposed HPLC methodology.

#### Accuracy and precision

To evaluate accuracy, the recovery percentages for each drug were calculated individually using their corresponding regression equations. The recovery rates fell within the range of 98.03% to 101.0%. The accuracy of the method for analyzing ternary mixtures was confirmed by calculating mean percentage recoveries with standard deviations from triplicate determinations (Table S1). The precision of the method was evaluated through triplicate measurements of each drug in three different ternary mixtures. Repeatability was determined by analyzing standard solutions containing the target drugs at three concentration levels within the linear range, with three replicate measurements performed within a single day. Intermediate precision was assessed by repeating the analysis at three concentrations (within the linear range) over three consecutive days, with triplicate measurements each day. The calculated percentage relative standard deviation (%RSD) for both repeatability and intermediate precision was found to be less than 2%, as presented in Table S2, demonstrating excellent method reproducibility.

#### Evaluation of analyte recovery from milk matrix

The analyte recovery (extraction efficiency) was evaluated by comparing the average peak area ratios of processed samples (0.05, 0.15, and 0.25 µg/mL) with those of corresponding non-extracted/authenticated standards. Quantitative analysis revealed extraction recoveries of 98.53% ± 0.63 for CFZ, 97.67% ± 1.09 for SDD, and 98.89% ± 0.58 for MFC, demonstrating excellent recovery rates across the studied concentration range. These results, presented in Table 3, indicate that the extraction methodology provides consistent and efficient recovery of all analytes.

**Table 3:** The extraction efficiency of the proposed sample Preparation methodology for the studied drugs.

	Drug Concentration (µg/mL)	Average peak area of processed sample	Average peak area of authenticated standard	% Recovery	Average percentage recoveries ± SD
CFZ	**0.05**	404.01 ± 3.17	413.22 ± 1.87	97.80	**98.53 ± 0.63**
	**0.15**	1207.77 ± 1.89	1220.32 ± 1.89	98.93	
	**0.25**	2027.91 ± 2.818	2050.02 ± 4.31	98.87	
SDD	**0.05**	193.547 ± 0.881	200.84 ± 5.26	96.50	**97.67 ± 1.09**
	**0.15**	723.827 ± 3.447	739.02 ± 2.35	97.83	
	**0.25**	1263.043 ± 7.744	1280.04 ± 3.14	98.67	
MFC	**0.05**	445.011 ± 3.17	453.2 ± 1.871	98.23	**98.89 ± 0.58**
	**0.15**	1497.77 ± 1.89	1510.32 ± 1.89	99.13	
	**0.25**	2503.22 ± 2.81	2520.02 ± 4.31	99.32	

#### Specificity and selectivity

Specificity and selectivity are critical parameters in analytical method validation as they indicate the method’s ability to measure the analyte accurately in the presence of other components, such as impurities, degradation products, or excipients that may be expected to be present. To evaluate the method’s specificity and selectivity, a comparative chromatographic analysis was performed using drug-free milk samples (blanks) alongside spiked and real milk samples. The results, presented in Fig. 3, showed no co-elution or signal overlap between endogenous matrix components and the target drugs, confirming the method’s ability to differentiate the analytes within a complex biological matrix. This high specificity and selectivity were mainly due to the optimized sample preparation process and the selection of a suitable detection wavelength. Milk is a complex matrix, composed of approximately 87.7% water, 4.9% lactose, 3.4% fat, 3.3% protein, and 0.7% minerals. Different types of milk (buffalo, cow, and goat) share the same composition but in different ratios. Our sample preparation procedure involved protein precipitation and fat removal through dispersive solid-phase extraction (dSPE) using Enhanced Matrix Removal-Lipid (EMR-L), which effectively minimized potential matrix interferences. Furthermore, detection was performed at 270 nm, a wavelength where carbohydrates and minerals do not absorb, and thereby further reducing matrix effects.

#### Robustness

The robustness of the method was evaluated by systematically investigating the effects of minor variations in key chromatographic parameters, such as flow rate, mobile phase pH, and column temperature, on the analytical response. These deliberate modifications were made to assess the method’s ability to withstand minor operational fluctuations. The study was carried out at different flow rates (1.00 ± 0.1 mL/min), various pH levels of the mobile phase (3.00 ± 0.2), and different column temperatures (25.00 ± 2 °C), as shown in Table S3. The data obtained showed consistent performance across the tested conditions, with %RSD values ranging from 1.23 to 1.97 (less than 2%) for all parameters. This thorough analysis confirms the method’s reliability and robustness under typical laboratory variations.

### Application on real milk samples

The proposed method was successfully applied to quantify the studied drug residues in cattle milk. The selected cattle included three lactating metritis-positive buffaloes and two lactating metritis-positive cows. They were receiving a combination therapy of the studied drugs in their recommended doses from a veterinarian. After a 72-hour withdrawal period following the last intramuscular dose, a 25 mL milk sample was collected individually from each lactating buffalo and cow. A total of five milk samples were collected, processed, and analyzed using the described chromatographic conditions. The concentrations in µg/mL were determined from the respective regression equations for each drug and compared to the Maximum Residual Limit (MRL) for each drug. The results are presented in Table 4, and Fig. 3 illustrates the HPLC chromatogram of sample no.1. All analyzed samples showed the presence of the studied drug residues within the acceptable MRL for each drug except for sample no. 4, which exhibited an SDD residual value above the MRL. Cow number 4, which was cured from metritis, should not be used for milk production until a complete clearance of SDD is achieved.

**Table 4:** Analysis of milk samples using the proposed method.

Milk samples	Sample type	Found Conc. (µg/mL)			MRL in milk (µg/mL)		
		CFZ	SDD	MFC	CFZ	SDD	MFC
Sample 1	Buffalo milk	0.013	0.014	–	0.05	0.1	0.15
Sample 2	Buffalo milk	–	–	0.052			
Sample 3	Buffalo milk	0.024	0.022	–			
Sample 4	Cow milk	–	0.205	–			
Sample 5	Cow milk	0.035	–	–			

### Evaluation of analyte stability in milk samples

The stability of the studied drugs in milk samples stored at -20 °C was evaluated by analyzing spiked milk samples at a concentration of 0.15 µg/mL over different storage time points (0, 1, 2, 3, and 6 weeks). The samples were considered stable if the nominal concentration remained within ± 15% (calculated as relative error, RE%). Results from Table S4 indicated that the RE% values were within ± 15% for up to two weeks of storage, demonstrating the stability of the drugs in milk at –20 °C during this period. However, after three weeks, the RE% values exceeded the specified range, and milk endogenous peaks were observed, some of which had the same retention times as the studied drugs.

### Statistical analysis

A comparative statistical evaluation between the developed analytical approach and previously published methods for the target analytes is summarized in Table 5. Owing to the unavailability of a mass spectrometer in our institution, the reported methods selected for comparison were HPLC-based rather than LC/MS. The experimental t-test and F-test values were consistently below their respective critical thresholds, demonstrating no statistically significant variance (*p* > 0.05) between the current methodology and established reference techniques. These findings confirm the equivalent accuracy and precision of the newly developed method relative to existing literature-reported procedures.

**Table 5:** Comparative statistical evaluation of the proposed and reported methods for the investigated drugs.

	CFZ		SDD		MFC	
	HPLC	Published method^23^	HPLC	Published method ^[16]^	HPLC	Published method^52^
Mean	100.39	97.1	99.08	94.6	98.66	96.21
± SD	1.00	4.87	0.39	6.54	0.75	3.53
% RSD	0.482	0.245	0.475	0.112	0.312	0.565
Variance	1	23.716	0.152	42.771	0.562	12.460
n	6	6	6	6	6	6
Student’s t- test	1.491 (2.202)^a^		1.675 (2.179)^a^		1.663 (2.202)^a^	
F-test	0.042 (6.09)^b^		0.003 (4.88)^b^		0.045 (6.09)^b^	

### The sustainability assessment of the developed method

**Fig. 4:**
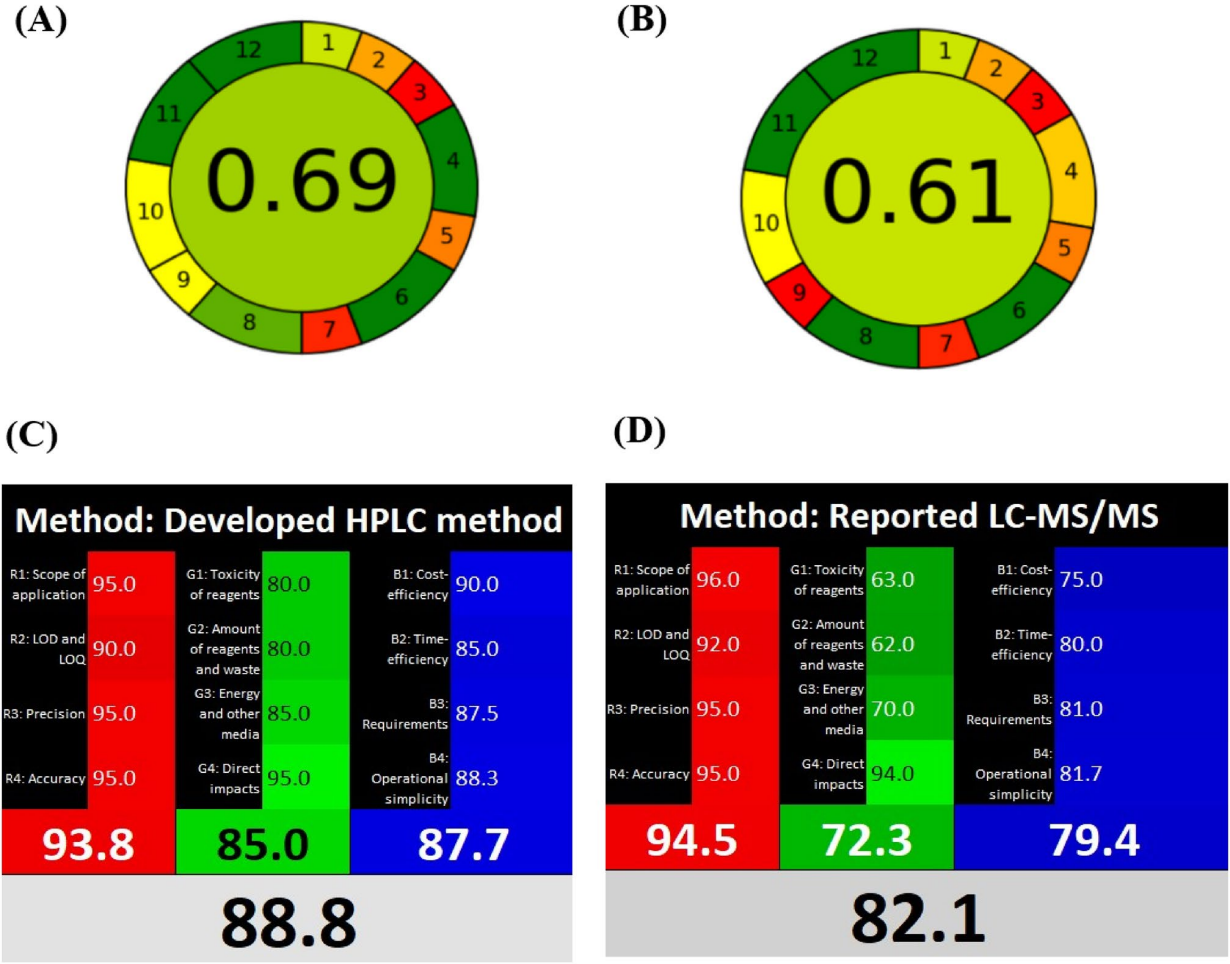
(**A **&** B**) AGREE evaluation of the developed HPLC method and the reported LC-MS/MS methods^71,72^, respectively. (**C **&** D**) RGB12 evaluation of the developed HPLC method and the reported LC-MS/MS methods^71,72^, respectively.

The sustainability of the developed analytical procedure was systematically evaluated using three assessment tools: the Analytical Eco-Scale^82,83^, AGREE^84^, and the RGB12 algorithm^85^ in comparison with the reported LC/MS methods^71,72^. The Eco-Scale approach employs a deductive scoring system (baseline = 100) that penalizes non-green aspects of the methodology, including hazardous reagent usage, energy requirements, waste generation, and operator safety concerns. Based on this evaluation, the developed method achieved an overall Eco-Scale score of 71, whereas the reported LC-MS/MS methods^71,72^ attained scores of 64 and 66, respectively (Table 6), highlighting the superior greenness of our approach. A complementary assessment was performed using the AGREE metric, which evaluates compliance with all 12 principles of Green Analytical Chemistry (GAC). The total score for the developed method was 0.69 (Fig. 4), while the reported methods achieved a total score of 0.61, confirming the method’s strong alignment with GAC principles. To provide a robust and comprehensive sustainability assessment, the Analytical Eco-Scale and AGREE metrics were integrated with the RGB12 algorithm to overcome the limitations of individual techniques. The RGB12 algorithm is a multi-criteria assessment protocol that is fully integrated with the principles of White Analytical Chemistry (WAC)^85^. WAC can be considered as an extension of the GAC, taking into account additional criteria apart from greenness. The RGB12 algorithm consists of 12 different algorithms divided into 3 groups: red, green, and blue. The red group focuses on important validation parameters such as scope of application, limit of detection, limit of quantitation, precision, and accuracy. The green group addresses the significant GAC parameters, including reagent toxicity and amounts, waste generation, energy management, and effects on animals and humans. The blue group pertains to cost efficiency, operational simplicity, time efficiency, and productivity. The RGB12 tool sums all scores from each group to define the final “whiteness” value, which reflects the method’s adherence to the WAC concept^85^. The developed method achieved an impressive whiteness score of 88.8, while the reported methods achieved a score of 82.1, as shown in Fig. 4. The RGB12 algorithm demonstrates that the developed HPLC method exhibited many advantages in terms of analytical efficiency, environmental friendliness, sustainability, economic feasibility, and practicality over the reported LC-MS/MS methods.

**Table 6:** Comparison of analytical Eco-Scale for the developed HPLC method and the reported LC/MS methods.

Parameters Reagents	Penalty Points (PPs)		
	The developed HPLC method	Reported LC-MS/MS Method^71^	Reported LC-MS/MS Method^72^
Type (No. of pictogram and signal word )	Potassium dihydrogen phosphate 0 Methanol 3*2 Acetonitrile 2*2	Methanol 3*2 Acetonitrile 2*2 Formic acid 3*2	Formic acid 3*2 Methanol 3*2
Amount (hazard* amount)	10–100 ml (12 + 8) Total of 20	10 to 100 ml 12 + 8 + 6 Total of 26	10 to 100 ml (12 + 12) Total of 24
Instrument			
Energy consumption	< 1.5 kW/h (1)	> 1.5 kW/h (2)	> 1.5 kW/h (2)
Emission of vapors or gasses	0	0	0
Waste			
Waste generated	> 10 mL (5)	> 10 mL (5)	> 10 mL (5)
Waste treatment	No treatment (3)	No treatment (3)	No treatment (3)
Total PPs	29	36	34
Score (100-PPs)	71	64	66

## Conclusion

CFZ, SDD, and MFC represent widely employed veterinary antimicrobial agents for infection control. Inappropriate administration practices or failure to observe proper withdrawal times may result in persistent drug residues in animal-derived food products, creating potential health hazards for consumers. The developed chromatographic technique offers a reliable, accurate, and reproducible solution for the quantitative determination of these pharmaceutical residues in dairy matrices, with complete elimination of matrix-related interferences. Comprehensive evaluation confirmed the method’s superior environmental profile, analytical performance, and operational advantages compared to conventional approaches, featuring enhanced green chemistry metrics, faster turnaround times, and cost-effective high-throughput capability for routine monitoring applications.

## Supplementary Information

Below is the link to the electronic supplementary material.

Supplementary Material 1

## Data Availability

All data generated or analyzed during this study are included in this published article [and its supplementary information files].

## References

[1] Beyene, T. Veterinary drug residues in Food-animal products: its risk factors and potential effects on public health. *J. Vet. Sci. Technol.* **7 **(1),285 (2015).

[2] Zhan, J. et al. ting, feng,. Generic and rapid determination of veterinary drug residues and other contaminants in raw milk by ultra performance liquid chromatography-tandem mass spectrometry. *J Chromatogr B Anal Technol Biomed Life Sci.* **906**, 48–57 (2012). 10.1016/j.jchromb.2012.08.018 22959038

[3] Turnipseed, S. B., Andersen, W. C., Karbiwnyk, C. M., Madson, M. R. & Miller, K. E. Multi-class, multi-residue liquid chromatography/tandem mass spectrometry screening and confirmation methods for drug residues in milk. *Rapid Commun. Mass. Spectrom.* **22** (10), 1467–1480 (2008). 18412094 10.1002/rcm.3532

[4] Delatour, T., Racault, L., Bessaire, T. & Desmarchelier, A. Screening of veterinary drug residues in food by LC-MS/MS. Background and challenges. *Food Addit. Contam. Part. A*. **35** (4), 633–646 (2018). 10.1080/19440049.2018.1426890 29324075

[5] Baynes, R. E. et al. Health concerns and management of select veterinary drug residues. *Food Chem. Toxicol.* **88**, 112–122 (2016). 26751035 10.1016/j.fct.2015.12.020

[6] Saleh, H., Elhenawee, M., Hussien, E. M., Ahmed, N. & Ibrahim, A. E. Validation of HPLC-UV Multi-Residue method for the simultaneous determination of Tetracycline, Oxytetracycline, spiramycin and neospiramycin in Raw milk. *Food Anal. Methods*. **14** (1), 36–43 (2021).

[7] Pratiwi, R., Ramadhanti, S. P., Amatulloh, A., Megantara, S. & Subra, L. Recent advances in the determination of veterinary drug residues in food. *Foods* **12** (18), 3422 (2023). 37761131 10.3390/foods12183422 PMC10527676

[8] Parmar, J. K., Singh, S., Gupta, V. & Kumar, U. Method optimization and validation of antibiotics residues in milk sample using LC-MS/MS. *Egypt. J. Vet. Sci.* **56**, 1–12 (2024).

[9] Ao, R. et al. Synthesis of COF (TAPA-DHTA)-sodium alginate-Ca2+-polyacrylate composite microspheres as adsorbent for dispersive solid phase extraction of fluoroquinolone antibiotics in food and water prior to the quantification by HPLC-MS/MS. *J. Chromatogr. A.*** 1757**, 466117 (2025). 10.1016/j.chroma.2025.466117 40494099

[10] Gao, P. et al. An optimized SPE-UPLC–MS/MS method for simultaneous quantification of 11 tetracyclines in dairy products. *J. Sci. Food Agric.* (2025). 10.1002/jsfa.70088 40728006

[11] Horvat, A. J. M. et al. Analysis, occurrence and fate of anthelmintics and their transformation products in the environment. *TrAC - Trends Anal. Chem.* **31**, 61–84 (2012).

[12] Celani, G. et al. Clinical efficacy of a single intravenous regional limb perfusion with Marbofloxacin versus ceftiofur sodium to treat acute interdigital phlegmon in dairy cows. *Animals* **13** (10), 1598 (2023). 37238027 10.3390/ani13101598 PMC10215901

[13] Nowacka-Kozak, E., Gajda, A. & Gbylik-Sikorska, M. Simultaneous determination of 68 antimicrobial compounds in pigs oral fluid by ultra-high performance liquid chromatography-tandem mass spectrometry. *J. Chromatogr. A*. **1729**, 465053 (2024). 38852267 10.1016/j.chroma.2024.465053

[14] Lopes, R. P., Augusti, D. V., Santos, F. A., Vargas, E. A. & Augusti, R. Development and validation of an efficient and innovative method for the quantification of multiclass veterinary drugs in milk by using LC–MS/MS analysis. *Anal. Methods*. **5** (19), 5121–5127 (2013).

[15] Sørensen, L. K. & Snor, L. K. Determination of cephalosporins in Raw bovine milk by high-performance liquid chromatography. *J. Chromatogr. A*. **882** (1–2), 145–151 (2000). 10895940 10.1016/s0021-9673(99)01317-5

[16] Tolika, E. P., Samanidou, V. F. & Papadoyannis, I. N. Development and validation of an HPLC method for the determination of ten sulfonamide residues in milk according to 2002/657/EC. *J. Sep. Sci.* **34** (14), 1627–1635 (2011). 21644254 10.1002/jssc.201100171

[17] Aresta, A., Cotugno, P. & Zambonin, C. Determination of ciprofloxacin, enrofloxacin, and Marbofloxacin in bovine urine, serum, and milk by Microextraction by a packed sorbent coupled to ultra-high performance liquid chromatography. *Anal. Lett.* **52** (5), 790–802 (2019).

[18] Woodward, K. N. Maximum residue limits. *Vet. Pharmacovigil. Advers React. Vet. Med. Prod.* 547–567 (2009).

[19] Grein, K. & Duarte, I. Establishing maximum residue limits in Europe. *Strateg Reducing Drug Chem. Residues Food Anim.* 49–63 (2014).

[20] Sher, N. et al. Novel HPLC method for quantitative determination of Cefazolin sodium in pharmaceutical formulations. *Res. Rep. Med. Chem.* **21, 21-28** (2013).

[21] Żandarek, J., Starek, M. & Dąbrowska, M. Development of thin-layer chromatography–densitometric procedure for qualitative and quantitative analyses and stability studies of Cefazolin. *Processes* **12** (3), 591 (2024).

[22] Piech, T., Majer-Dziedzic, B., Kostruba, A., Grzelak, E. M. & Choma, I. M. Thin-layer chromatography—Direct bioautography as an alternative method for screening of antibiotic residues in milk: A comparative study. *J. Liq Chromatogr. Relat. Technol.* **39** (5–6), 292–297 (2016).

[23] Karageorgou, E. G. & Samanidou, V. F. Application of ultrasound-assisted matrix solid-phase dispersion extraction to the HPLC confirmatory determination of cephalosporin residues in milk. *J. Sep. Sci.* **33** (17–18), 2862–2871 (2010). 20715145 10.1002/jssc.201000385

[24] Cámara, M. et al. An HPLC-DAD method for the simultaneous determination of nine β-lactam antibiotics in Ewe milk. *Food Chem.* **141** (2), 829–834 (2013). 23790854 10.1016/j.foodchem.2013.02.131

[25] Kantiani, L., Farré, M. & Barceló, D. Analytical methodologies for the detection of β-lactam antibiotics in milk and feed samples. *TrAC Trends Anal. Chem.* **28** (6), 729–744 (2009).

[26] Moudgil, P., Bedi, J. S., Aulakh, R. S., Gill, J. P. S. & Kumar, A. Validation of HPLC multi-residue method for determination of fluoroquinolones, tetracycline, sulphonamides and Chloramphenicol residues in bovine milk. *Food Anal. Methods*. **12**, 338–346 (2019).

[27] Karageorgou, E. G., Samanidou, V. F. & Papadoyannis, I. N. Ultrasound-assisted matrix solid phase dispersive extraction for the simultaneous analysis of β‐lactams (four penicillins and eight cephalosporins) in milk by high performance liquid chromatography with photodiode array detection. *J. Sep. Sci.* **35** (19), 2599–2607 (2012). 22941669 10.1002/jssc.201200514

[28] Liu, X. et al. Solid phase extraction using magnetic core mesoporous shell microspheres with C18-modified interior pore-walls for residue analysis of cephalosporins in milk by LC–MS/MS. *Food Chem.* **150**, 206–212 (2014). 24360441 10.1016/j.foodchem.2013.10.145

[29] Daeseleire, E., Ruyck, H. & Van De, Renterghem, R. Confirmatory assay for the simultaneous detection of penicillins and cephalosporins in milk using liquid chromatography/tandem mass spectrometry. *Rapid Commun. mass. Spectrom.* **14** (15), 1404–1409 (2000). 10920362 10.1002/1097-0231(20000815)14:15<1404::AID-RCM38>3.0.CO;2-4

[30] Holstege, D. M., Puschner, B., Whitehead, G. & Galey, F. D. Screening and mass spectral confirmation of β-lactam antibiotic residues in milk using LC-MS/MS. *J. Agric. Food Chem.* **50** (2), 406–411 (2002). 11782216 10.1021/jf010994s

[31] Junza, A., Amatya, R., Barrón, D. & Barbosa, J. Comparative study of the LC–MS/MS and UPLC–MS/MS for the multi-residue analysis of quinolones, penicillins and cephalosporins in cow milk, and validation according to the regulation 2002/657/EC. *J. Chromatogr. B*. **879** (25), 2601–2610 (2011). 10.1016/j.jchromb.2011.07.018 21820979

[32] Jank, L. et al. High-throughput method for the determination of residues of β-lactam antibiotics in bovine milk by LC-MS/MS. *Food Addit. Contam. Part. A*. **32** (12), 1992–2001 (2015). 10.1080/19440049.2015.1099745 26414060

[33] Meklati, F. R. et al. Comparative assessment of antibiotic residues using liquid chromatography coupled with tandem mass spectrometry (LC-MS/MS) and a rapid screening test in Raw milk collected from the North-Central Algerian dairies. *Toxics* **10** (1), 19 (2022). 35051061 10.3390/toxics10010019 PMC8781432

[34] Karageorgou, E., Myridakis, A., Stephanou, E. G. & Samanidou, V. Multiresidue LC–MS/MS analysis of cephalosporins and quinolones in milk following ultrasound-assisted matrix solid‐phase dispersive extraction combined with the quick, easy, cheap, effective, rugged, and safe methodology. *J. Sep. Sci.* **36** (12), 2020–2027 (2013). 23568854 10.1002/jssc.201300194

[35] Li, W. et al. Simultaneous determination of 22 cephalosporins drug residues in pork muscle using liquid chromatography–tandem mass spectrometry. *J. Chromatogr. B*. **1022**, 298–307 (2016). 10.1016/j.jchromb.2016.04.026 27131893

[36] Rezende, C. P., Almeida, M. P., Brito, R. B., Nonaka, C. K. & Leite, M. O. Optimisation and validation of a quantitative and confirmatory LC-MS method for multi-residue analyses of β-lactam and Tetracycline antibiotics in bovine muscle. *Food Addit. Contam. Part. A*. **29** (4), 541–549 (2012). 10.1080/19440049.2011.627883 22070766

[37] Sattar, O. I. A., Abuseada, H. H. M., Emara, M. S. & Rabee, M. Eco-friendly multivariate curve resolution-alternating least squares and chromatographic quantifications of some veterinary drug residues in pharmaceutical industrial wastewater. *RSC Adv.* **11** (5), 2935–2946 (2021). 35424235 10.1039/d0ra08850a PMC8693979

[38] Van Poucke, L. S. G., Depourcq, G. C. I. & Van Peteghem, C. H. A quantitative method for the detection of sulfonamide residues in meat and milk samples with a high-performance thin-layer chromatographic method. *J. Chromatogr. Sci.* **29** (10), 423–427 (1991). 1808214 10.1093/chromsci/29.10.423

[39] Unruh, J., Piotrowski, E., Schwartz, D. P. & Barford, R. Solid-phase extraction of sulfamethazine in milk with quantitation at low Ppb levels using thin-layer chromatography. *J. Chromatogr. A*. **519** (1), 179–187 (1990).

[40] Kanda, M. et al. Development and performance evaluation of a Microbiological method for screening and LC-MS/MS for conformation of sulfonamides in animal-derived foods. *Food Addit. Contam. Part. A*. **41** (8), 900–913 (2024). 10.1080/19440049.2024.2368903 38913845

[41] Wen, Y., Zhang, M., Zhao, Q. & Feng, Y. Q. Monitoring of five sulfonamide antibacterial residues in milk by in-tube solid-phase Microextraction coupled to high-performance liquid chromatography. *J. Agric. Food Chem.* **53** (22), 8468–8473 (2005). 16248539 10.1021/jf051319b

[42] Wu, Y. L., Li, C., Liu, Y. J. & Shen, J. Z. Validation method for the determination of sulfonamide residues in bovine milk by HPLC. *Chromatographia* **66**, 191–195 (2007).

[43] Samanidou, V. F., Tolika, E. P. & Papadoyannis, I. N. Development and validation of an HPLC confirmatory method for the residue analysis of four sulphonamides in cow’s milk according to the European union decision 2002/657/EC. *J. Liq Chromatogr. Relat. Technol.* **31** (9), 1358–1372 (2008).

[44] Granja, R. H. M. M., de Lima, A. C., Salerno, A. G. & Wanschel, A. C. B. A. Validation of a liquid chromatography with ultraviolet detection methodology for the determination of sulfonamides in bovine milk according to 2002/657/EC. *Food Control*. **28** (2), 304–308 (2012).

[45] Yang, T. C., Yang, I. & Liao, L. Determination of sulfonamide residues in milk by on-line Microdialysis and HPLC. *J. Liq Chromatogr. Relat. Technol.* **27** (3), 501–510 (2004).

[46] Agarwal, V. K. Detection of sulfamethazine residues in milk by high performance liquid chromatography. *J. Liq Chromatogr.* **13** (17), 3531–3539 (1990).

[47] Gamba, V. et al. Development and validation of a confirmatory method for the determination of sulphonamides in milk by liquid chromatography with diode array detection. *Anal. Chim. Acta*. **637** (1–2), 18–23 (2009). 19286007 10.1016/j.aca.2008.09.022

[48] Lavrukhina, O. I., Amelin, V. G., Kish, L. K., Tretyakov, A. V. & Pen’kov, T. D. Determination of residual amounts of antibiotics in environmental samples and food products. *J. Anal. Chem.* **77** (11), 1349–1385 (2022).

[49] Samanidou, V. F., Tolika, E. P. & Papadoyannis, I. N. Chromatographic residue analysis of sulfonamides in foodstuffs of animal origin. *Sep. Purif. Rev.* **37** (4), 325–371 (2008).

[50] Vílchez, J. L., Navalón, A., Araujo, L. & Prieto, A. Determination of Danofloxacin and Marbofloxacin in milk samples by micellar liquid chromatography with fluorescence detection. *Anal. Lett.* **40** (3), 601–613 (2007).

[51] Karami-Osboo, R., Shojaee, M. H., Miri, R., Kobarfard, F. & Javidnia, K. Simultaneous determination of six fluoroquinolones in milk by validated QuEChERS-DLLME HPLC-FLD. *Anal. Methods*. **6** (15), 5632–5638 (2014).

[52] Navrátilová, P., Borkovcová, I., Vyhnálková, J. & Vorlová, L. Fluoroquinolone residues in Raw cow’s milk. *Czech J. Food Sci.* **29** (6), 641 (2011).

[53] da Silva, T. L. A., Ferreira, R. G. L., Lustosa, I. A. & Kogawa, A. C. An overview of analytical methods for the quantification of Marbofloxacin in Pharmaceutical, Biological, and food matrixes. *J. AOAC Int.* **105** (2), 456–462 (2022). 34718603 10.1093/jaoacint/qsab143

[54] Abdel Aziz, E. A., El-Nabtity, S. M., El Barawy, A. A. M. & Saleh, M. A. M. Determination of Marbofloxacin residues in rabbit tissues by HPLC. *Zagazig Vet. J.* **45** (1), 39–46 (2017).

[55] Marazuela, M. D. & Moreno-Bondi, M. C. Multiresidue determination of fluoroquinolones in milk by column liquid chromatography with fluorescence and ultraviolet absorbance detection. *J. Chromatogr. A*. **1034** (1–2), 25–32 (2004). 15116911 10.1016/j.chroma.2004.02.022

[56] Christodoulou, E. A. & Samanidou, V. F. Multiresidue HPLC analysis of ten quinolones in milk after solid phase extraction: validation according to the European union decision 2002/657/EC. *J. Sep. Sci.* **30** (15), 2421–2429 (2007). 17683042 10.1002/jssc.200700129

[57] Rodríguez-Díaz, R. C., Fernández-Romero, J. M., Aguilar-Caballos, M. P. & Gómez-Hens, A. Determination of fluoroquinolones in milk samples by postcolumn derivatization liquid chromatography with luminescence detection. *J. Agric. Food Chem.* **54** (26), 9670–9676 (2006). 17177486 10.1021/jf0621368

[58] Martins-Júnior, H. A., Kussumi, T. A., Wang, A. Y. & Lebre, D. T. A rapid method to determine antibiotic residues in milk using liquid chromatography coupled to electrospray tandem mass spectrometry. *J. Braz Chem. Soc.* **18**, 397–405 (2007).

[59] Jank, L. et al. Liquid chromatography–tandem mass spectrometry multiclass method for 46 antibiotics residues in milk and meat: development and validation. *Food Anal. Methods*. **10**, 2152–2164 (2017).

[60] Van Hoof, N. Multi-residue LC-MS method for the detection of quinolones in muscle and bovine milk. Dev LC-MS n methods residue-analysis. *Vet. Med. Prod.* **529**, 265–272 (2005).

[61] Liu, Y. et al. Current analytical strategies for the determination of quinolone residues in milk. *Food Chem.* **430**, 137072 (2024). 37549624 10.1016/j.foodchem.2023.137072

[62] Aguilera-Luiz, M. M., Vidal, J. L. M., Romero-González, R. & Frenich, A. G. Multi-residue determination of veterinary drugs in milk by ultra-high-pressure liquid chromatography–tandem mass spectrometry. *J. Chromatogr. A*. **1205** (1–2), 10–16 (2008). 18752803 10.1016/j.chroma.2008.07.066

[63] Hermo, M. P., Nemutlu, E., Kır, S., Barrón, D. & Barbosa, J. Improved determination of quinolones in milk at their MRL levels using LC–UV, LC–FD, LC–MS and LC–MS/MS and validation in line with regulation 2002/657/EC. *Anal Chim Acta.* **613 **(1), 98–107 (2008). 10.1016/j.aca.2008.02.045 18374707

[64] Zhang, C., Wang, H., Mu, Y. & Liu, H. A method for simultaneously and accurately quantify seven quinolones in matrix reference materials by HPLC-MS. *Microchem J.* **204**, 110984 (2024).

[65] Luo, M. et al. Sensitive immunoassays based on a monoclonal antibody for detection of Marbofloxacin in milk. *J. Dairy. Sci.* **103** (9), 7791–7800 (2020). 32684479 10.3168/jds.2019-18108

[66] Yang, X. et al. An immunochromatographic strip sensor for Marbofloxacin residues. *PLoS One*. **19** (3), e0299709 (2024). 38551994 10.1371/journal.pone.0299709 PMC10980191

[67] Chen, X. et al. Simultaneous screening for Marbofloxacin and Ofloxacin residues in animal-derived foods using an indirect competitive immunoassay. *Food Agric. Immunol.* **28** (3), 489–499 (2017).

[68] Sheng, W. et al. Determination of Marbofloxacin residues in beef and pork with an enzyme-linked immunosorbent assay. *J. Agric. Food Chem.* **57** (13), 5971–5975 (2009). 19522498 10.1021/jf900940n

[69] Xiao, J. et al. Rapid detection of fluoroquinolone residues in aquatic products based on a gold-labeled microwell immunochromatographic assay. *Food Qual. Saf.* **6**, fyac033 (2022).

[70] Pinacho, D. G., Sánchez-Baeza, F., Pividori, M. I. & Marco, M. P. Electrochemical detection of fluoroquinolone antibiotics in milk using a Magneto immunosensor. *Sensors* **14** (9), 15965–15980 (2014). 25171120 10.3390/s140915965 PMC4208156

[71] Robert, C. et al. Rapid multiresidue and multi-class screening for antibiotics and benzimidazoles in feed by ultra high performance liquid chromatography coupled to tandem mass spectrometry. *Food Control*. **50**, 509–515 (2015). 10.1080/19440049.2016.1207808 27376829

[72] Varenina, I., Bilandžić, N., Luburić, D. J., Božić, Kolanović, B. S. & Varga, I. High resolution mass spectrometry method for the determination of 13 antibiotic groups in bovine, swine, poultry and fish meat: an effective screening and confirmation analysis approach for routine laboratories. *Food Control*. **133**, 108576 (2022).

[73] Buchberger, W. W. Novel analytical procedures for screening of drug residues in water, waste water, sediment and sludge. *Anal. Chim. Acta*. **593**, 129–139 (2007). 17543599 10.1016/j.aca.2007.05.006

[74] Magon, T. et al. Simultaneous determination of four antibiotics in Raw milk by UPLC-MS/MS using protein precipitation as sample preparation: Development, validation, and application in real samples. *J. Braz Chem. Soc.* **29** (11), 2441–2448 (2018).

[75] Castilla-Fernández, D., Moreno-González, D., Beneito-Cambra, M. & Molina-Díaz, A. Critical assessment of two sample treatment methods for multiresidue determination of veterinary drugs in milk by UHPLC-MS/MS. *Anal. Bioanal Chem.* **411**, 1433–1442 (2019). 30683965 10.1007/s00216-019-01582-y

[76] Luo, P. et al. Simultaneous determination of 169 veterinary drugs in chicken eggs with EMR-Lipid clean-up using ultra-high performance liquid chromatography tandem mass spectrometry. *Anal. Methods*. **11** (12), 1657–1662 (2019).

[77] Du Sert, N. P. et al. The ARRIVE guidelines 2.0: Updated guidelines for reporting animal research. (2020). 10.1371/journal.pbio.3000410 PMC7360023 32663219

[78] Ghaffari, S., Shahrouzi, J. R., Towfighi, F. & Khoshfetrat, A. B. Partitioning of Cefazolin in aqueous two-phase systems containing Poly (ethylene glycol) and sodium salts (citrate, tartrate, and sulphate). *Fluid Phase Equilib.* **488**, 54–61 (2019).

[79] Elbadawy, M., Ishihara, Y., Aboubakr, M., Sasaki, K. & Shimoda, M. Oral absorption profiles of sulfonamides in Shiba goats: a comparison among sulfadimidine, sulfadiazine and sulfanilamide. *J. Vet. Med. Sci.* **78** (6), 1025–1029 (2016). 27010464 10.1292/jvms.15-0601 PMC4937137

[80] Fernández-Varón, E. et al. PK/PD analysis of Marbofloxacin by Monte Carlo simulation against Mycoplasma agalactiae in plasma and milk of lactating goats after IV, SC and SC-long acting formulations administration. *Animals* **11** (4), 1104 (2021). 33921496 10.3390/ani11041104 PMC8069869

[81] Guideline, I. C. H. H. T. others. Validation of analytical procedures: text and methodology. *Q2* **1** (20), 5 (2005).

[82] Gałuszka, A., Migaszewski, Z. M., Konieczka, P. & Namieśnik, J. Analytical Eco-Scale for assessing the greenness of analytical procedures. *TrAC Trends Anal. Chem.* **37**, 61–72 (2012).

[83] Hammad, S. F., Hamid, M. A. A., Adly, L. & Elagamy, S. H. Comprehensive review of Greenness, Whiteness, and blueness assessments of analytical methods. *Green. Anal. Chem.* **12,** 100209**,** (2025).

[84] Pena-Pereira, F., Wojnowski, W. & Tobiszewski, M. AGREE—Analytical greenness metric approach and software. *Anal. Chem.* **92** (14), 10076–10082 (2020). 32538619 10.1021/acs.analchem.0c01887 PMC7588019

[85] Yin, L. et al. Green analytical chemistry metrics for evaluating the greenness of analytical procedures. *J. Pharm. Anal.* 101013 (2024). 10.1016/j.jpha.2024.101013 PMC11697060 39759968

