# Supplementary material for: Sustainable HPLC method for simultaneous determination of Cefazolin, Sulfadimidine, and Marbofloxacin residues in milk
Source: Sci Rep. 2026 Jan 6;16:793. doi: 10.1038/s41598-025-32613-7 (PMC12780186; doi:10.1038/s41598-025-32613-7)
Supplement: Supplementary file 1 — Supplementary Material 1 [file 41598_2025_32613_MOESM1_ESM.docx]

**Supplementary information**

**
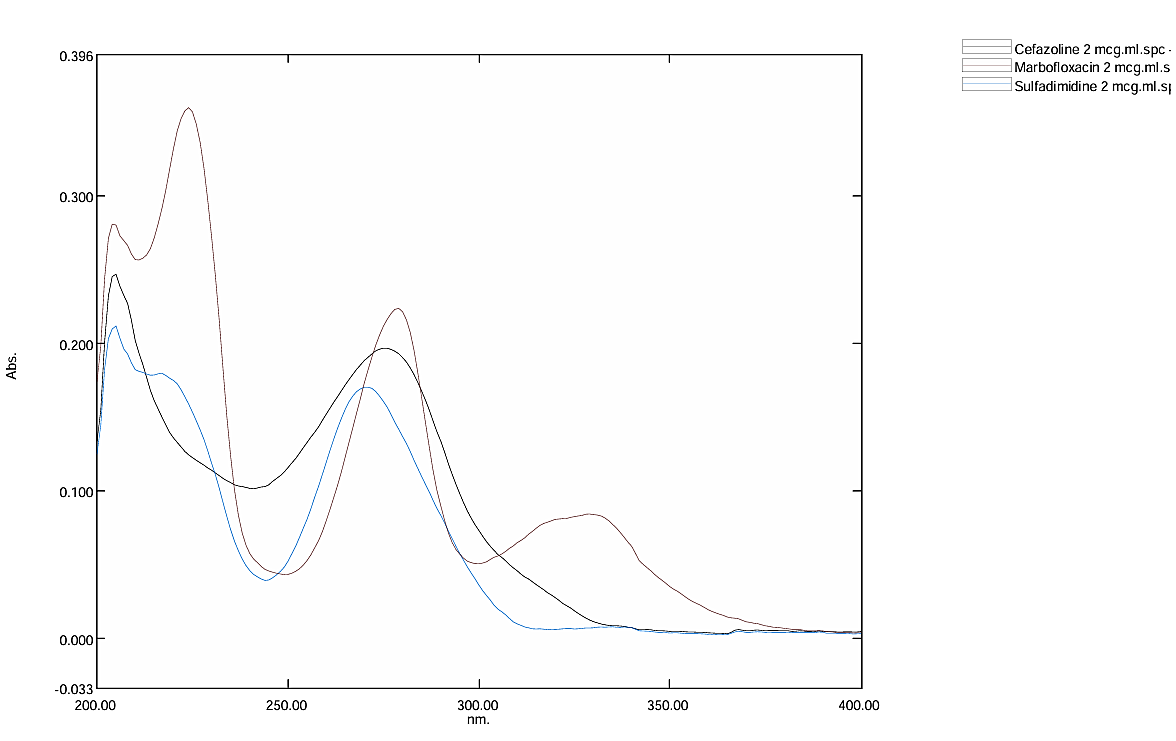
**

**Figure S1: Overlay spectra of CFZ, SDD, MFC**

**Table S1: Results of accuracy for determination of Cefazolin (CFZ), Sulfadimidine (SDD), and Marbofloxacin (MFC) by the proposed HPLC method**

|  | **Conc. (µg/mL)** | **Mean conc. Found ^A^ (µg/mL)** | **% Recovery** | **Mean % recovery** ± **SD** |
| --- | --- | --- | --- | --- |
| **CFZ** | 0.05 | 0.051 | 100.31 | 99.38 ± 0.82 |
|  | 0.15 | 0.148 | 98.76 |  |
|  | 0.25 | 0.247 | 99.08 |  |
| **SDD** | 0.05 | 0.049 | 99.02 | 99.21 ± 0.16 |
|  | 0.15 | 0.148 | 99.33 |  |
|  | 0.25 | 0.249 | 99.27 |  |
| **MFC** | 0.05 | 0.049 | 99.80 | 99.32 ± 0.77 |
|  | 0.15 | 0.149 | 99.74 |  |
|  | 0.25 | 0.246 | 98.43 |  |

***^A^*** Average of three determinations.

**Table S2: Evaluation of precision study for determination of Cefazolin (CFZ), Sulfadimidine (SDD), and Marbofloxacin (MFC) by the proposed HPLC method**

|  | **Conc. (µg/mL)** | **Intra-day (Repeatability)** | | | **Inter-day (Intermediate precision)** | | | **Pooled RSD (%)** |
| --- | --- | --- | --- | --- | --- | --- | --- | --- |
|  |  | **Mean conc. Found (µg/mL)** | **Mean % recovery ^A^** ± **SD** | **RSD (%)** | **Mean conc. Found (µg/mL)** | **Mean % recovery ^A^** ± **SD** | **RSD (%)** |  |
| **CFZ** | 0.05 | 0.051 | 100.31 ± 0.328 | 0.228 | 0.050 | 100.76 ± 0.214 | 0.254 | 0.377 |
|  | 0.15 | 0.148 | 98.76 ± 0.282 | 0.219 | 0.148 | 99.23 ± 0.247 | 0.475 |  |
|  | 0.25 | 0.247 | 99.08 ± 0.437 | 0.521 | 0.248 | 99.17 ± 0.135 | 0.567 |  |
| **SDD** | 0.05 | 0.049 | 99.02 ± 0.129 | 0.139 | 0.049 | 99.45 ± 0.423 | 0.147 | 0.274 |
|  | 0.15 | 0.148 | 99.33 ± 0.301 | 0.284 | 0.149 | 99.42 ± 0.139 | 0.252 |  |
|  | 0.25 | 0.249 | 99.27 ± 0.403 | 0.467 | 0.249 | 99.86 ± 0.427 | 0.352 |  |
| **MFC** | 0.05 | 0.049 | 99.80 ± 0.342 | 0.783 | 0.049 | 97.47 ± 0.522 | 0.325 | 0.401 |
|  | 0.15 | 0.149 | 99.74 ± 0.207 | 0.236 | 0.149 | 99.48 ± 0.132 | 0.333 |  |
|  | 0.25 | 0.246 | 98.43 ± 0.172 | 0.273 | 0.245 | 98.19 ± 0.573 | 0.452 |  |

***^A^*** Average of three determinations.

**Table S3: Robustness results of the proposed method**

| **Parameter** | **Exp. Change** | **CFZ** | | | **SDD** | | | **MFC** | | |
| --- | --- | --- | --- | --- | --- | --- | --- | --- | --- | --- |
|  |  | **N ^a^** | **T ^b^** | **K ^c^** | **N ^a^** | **T ^b^** | **K ^c^** | **N ^a^** | **T ^b^** | **K ^c^** |
| **Flow rate (mL/min)** | 1.00 + 0.1 | 11718 | 0.83 | 1.74 | 10459 | 0.98 | 3.89 | 12143 | 1.11 | 4.72 |
|  | 1.00 - 0.1 | 11768 | 0.84 | 1.78 | 10395 | 0.96 | 3.89 | 12261 | 1.12 | 4.87 |
| **pH value (unit)** | 3.00 + 0.2 | 11938 | 0.83 | 1.83 | 10568 | 0.93 | 3.74 | 12359 | 1.13 | 4.90 |
|  | 3.00 - 0.2 | 11699 | 0.83 | 1.83 | 10419 | 0.95 | 3.76 | 12083 | 1.12 | 4.89 |
| **Column Temp. (°C)** | 25.00 + 2 | 11820 | 0.81 | 1.79 | 10450 | 0.96 | 3.74 | 12110 | 1.09 | 4.77 |
|  | 25.00 - 2 | 11887 | 0.81 | 1.82 | 10625 | 0.94 | 3.77 | 12207 | 1.11 | 4.87 |
|  | **Mean** | 11805 | 0.82 | 1.80 | 10486 | 0.95 | 3.80 | 12193 | 1.11 | 4.84 |
|  | **SD** | 94.6 | 0.01 | 0.04 | 90.3 | 0.02 | 0.07 | 103.7 | 0.01 | 0.07 |
|  | **% RSD** | 1.97 | 1.48 | 1.97 | 1.65 | 1.84 | 1.87 | 1.68 | 1.23 | 1.53 |

***^a^*** Number of theoretical plates.

**^b^** Tailing factor.

**^c^** Capacity factor.

**Table S4: Evaluation of analyte stability in milk samples at storage condition of -20 °C**

| **Storage time points** | **CFZ**  **(0.15 µg/mL)** | | **SDD**  **(0.15 µg/mL)** | | **MFC**  **(0.15 µg/mL)** | |
| --- | --- | --- | --- | --- | --- | --- |
|  | **Found Conc. (µg/mL)** | **RE% ^a^** | **Found Conc. (µg/mL)** | **RE% ^a^** | **Found Conc. (µg/mL)** | **RE% ^a^** |
| Zero time | 0.148 | -1.33 | 0.149 | -0.67 | 0.151 | 0.66 |
| After 1 week | 0.146 | -2.67 | 0.147 | -2.00 | 0.146 | -2.67 |
| After 2 weeks | 0.142 | -5.34 | 0.148 | -1.34 | 0.142 | -5.34 |
| After 3 weeks | 0.095 | -36.67 | 0.106 | -29.34 | 0.112 | -25.34 |
| After 6 weeks | 0.032 | -78.67 | 0.056 | -62.67 | 0.068 | -54.67 |

^A^ RE% is the relative error percentage.
